# Feasibility and diagnostic accuracy of point-of-care handheld echocardiography in acute ischemic stroke patients – a pilot study

**DOI:** 10.1186/s12883-017-0937-8

**Published:** 2017-08-11

**Authors:** Peter Kraft, Anna Fleischer, Silke Wiedmann, Viktoria Rücker, Daniel Mackenrodt, Caroline Morbach, Uwe Malzahn, Christoph Kleinschnitz, Stefan Störk, Peter U. Heuschmann

**Affiliations:** 1 Department of Neurology, University Hospital of Würzburg, Josef-Schneider-Str. 11, 97080 Würzburg, Germany; 2 Institute of Clinical Epidemiology and Biometry, University of Würzburg, Würzburg, Germany; 3 Comprehensive Heart Failure Center, University of Würzburg, Würzburg, Germany; 4 Department of Neurology, University Hospital Essen, Essen, Germany; 5 Department of Internal Medicine, University Hospital Würzburg, Würzburg, Germany

**Keywords:** Ischemic stroke, Point-of-care echocardiography, Ejection fraction, Stroke unit, Systolic dysfunction, Feasibility, Accuracy

## Abstract

**Background:**

Standard echocardiography (SE) is an essential part of the routine diagnostic work-up after ischemic stroke (IS) and also serves for research purposes. However, access to SE is often limited. We aimed to assess feasibility and accuracy of point-of-care (POC) echocardiography in a stroke unit (SU) setting.

**Methods:**

IS patients were recruited on the SU of the University Hospital Würzburg, Germany. Two SU team members were trained in POC echocardiography for a three-month period to assess a set of predefined cardiac parameters including left ventricular ejection fraction (LVEF). Diagnostic agreement was assessed by comparing POC with SE executed by an expert sonographer, and intraclass correlation coefficient (ICC) or kappa (κ) with 95% confidence intervals (95% CI) were calculated.

**Results:**

In the 78 patients receiving both POC and SE agreement for cardiac parameters was good, with ICC varying from 0.82 (95% CI 0.71–0.89) to 0.93 (95% CI 0.87–0.96), and κ from 0.39 (−95% CI 0.14–0.92) to 0.79 (95% CI 0.67–0.91). Detection of systolic dysfunction with POC echocardiography compared to SE was very good, with an area under the curve of 0.99 (0.96–1.00). Interrater agreement for LVEF measured by POC echocardiography was good with κ 0.63 (95% CI 0.40–0.85).

**Conclusions:**

POC echocardiography in a SU setting is feasible enabling reliable quantification of LVEF and preliminary assessment of selected cardiac parameters that might be used for research purposes. Its potential clinical utility in triaging stroke patients who should undergo or do not necessarily require SE needs to be investigated in larger prospective diagnostic studies.

**Electronic supplementary material:**

The online version of this article (doi:10.1186/s12883-017-0937-8) contains supplementary material, which is available to authorized users.

## Background

About 30% of ischemic strokes are attributable to cardiac causes such as atrial fibrillation, patent foramen ovale, or other structural or rhythm abnormalities associated with systolic dysfunction [1]. In patients without known atrial fibrillation, echocardiography is considered essential to determine the underlying cause of stroke in research studies and for guiding secondary prevention in clinical routine; its use, therefore, is recommended by current clinical guidelines [2–4]. However, access of stroke patients to echocardiography that is executed by an ultrasound specialist is often limited, which may delay diagnosis, extend hospital stay and increase healthcare costs [5]. Point-of-care (POC) echocardiography (so-called ‘handheld echo’) might be used in stroke patients as a screening tool for the detection of treatment relevant cardiac abnormalities, such as systolic dysfunction, aortic and mitral valve disease, left ventricular hypertrophy, atrial septal aneurysm, and pleural and pericardial effusion. In addition, POC echocardiography could contribute valuable information needed for research purposes, such as stroke etiology in diagnostic studies.

In this pilot study we investigated the diagnostic utility and accuracy of POC echocardiography in acute stroke patients performed by a non-expert examiner after a defined training phase on a stroke unit compared to a state-of-the-art transthoracic echocardiography performed by an expert sonographer under standardized cardiological conditions.

## Methods

### Population

Patients were recruited consecutively on the stroke unit of the University Hospital Würzburg between May and August 2014. The patient group is a sub-sample of the prospective SICFAIL (Stroke Induced Cardiac FAILure) cohort study evaluating the prevalence and time course of cardiac dysfunction in patients with ischemic stroke. Inclusion criteria were a diagnosis of acute ischemic stroke according to world health organization (WHO) definition [6], age ≥ 18 years, and written informed consent.

### Echocardiography

POC echocardiography was performed using a portable cardiac ultrasound device (Vivid q, GE Healthcare) equipped with a M3S 1.5–4.0 MHz scanner (Additional file 1: Figure S1) in all study participants by a medical student (AF) specifically trained in this technique for the purpose of this study. A neurologist unfamiliar with echocardiography prior to study start (PK) received similar training to quantify inter-rater reliability. Training of the two non-cardiologist sonographers (AF, PK) was provided by expert sonographers of the Comprehensive Heart Failure Center Würzburg, spanned a period of about 3 months, included POC and standard echocardiography, and was completed via 3 certification scans 2 months prior to study initiation. For POC echocardiography measurements, patients were examined lying left-sided in their regular stroke unit beds, i.e. not on a dedicated echocardiography stretcher. The left arm had to be bended behind the head and the upper part of the bed was elevated by 20–30° to the horizontal plane. The patients had to follow the examiner’s breathing instructions as far as possible. The examination started with the parasternal and continued with the apical and finally the subcostal insonation.

In addition, all patients underwent a reference transthoracic standard echocardiography according to current guidelines performed on a high-end ultrasound device (Vivid E9, GE Healthcare; GE M5S-D matrix single-crystal phased array transducer) by an expert sonographer of the Comprehensive Heart Failure Center [7].

In every patient 3 electrocardiography-triggered heart cycles were stored for both the POC and standard echocardiography. Basic cardiac parameters were assessed by standard scanning procedures including left ventricular ejection fraction (LVEF), left heart dimensions, wall thickness, major valve abnormalities, and pericardial or pleural effusion. LVEF was calculated according to the Simpson’s biplane method or, in case of suboptimal imaging conditions, according to the Simpson’s monoplane method. An LVEF <55% was defined as systolic dysfunction. Both scans, POC and standard echocardiography were performed within 48 h. In every case, the examiner performing the POC test vs. the standard echocardiography was blinded to the results of the respectively other examination.

### Statistics and sample size calculation

For continuous variables the mean and standard deviation, for categorical variables the frequencies and proportions were reported. To assess the agreement between the POC and standard echocardiography for qualitative variables, Cohen’s kappa with its respective 95% confidence interval (CI) was calculated. To assess the agreement between the POC and standard echocardiography for quantitative variables in the sense of conformity with standard echocardiography as reference, the intraclass correlation coefficient (ICC) for two raters (for continuous variables) with their respective 95% CI were calculated.

The sample size estimation was based on LVEF as the main read-out parameter as the SICFAIL study focused on echocardiographic measurement of cardiac function defined by ejection fraction. Area under the receiver operation curve (AUC), sensitivity and specificity were assessed for the detection of systolic dysfunction with POC echocardiography; 78 were required to detect an AUC of 0.95 with the power of 80% to the significance level of 5%. Analyses were performed with SPSS Software (SPSS 23, IBM Corp.).

### Ethical approval

The study was approved from the University Hospital Würzburg Ethics Committee (AZ 176/13) and patients provided their written consent to participate in the study prior to any examination. The study was reported according to the Standards for Reporting Diagnostic accuracy studies (STARD) criteria.

## Results

Eighty one patients with ischemic stroke were recruited for this study. Three subjects (3.7%) were excluded from analyses as they refused subsequent standard echocardiography. Hence, 78 patients provided two echocardiograms. In 76% of cases both echocardiographies were conducted within 24 h, in 24% within 48 h*.* In 56 patients (72%) standard echocardiography followed POC examination, in 22 patients (28%) POC echocardiography was conducted first. The demographic and clinical baseline characteristics are shown in Table 1.

**Table 1:** Demographical and baseline clinical data of patients

Age, years, mean (SD)	68.0 (13.5)
Male, n (%)	47 (61.0)
Hypertension, n (%)	44 (58.7)
Diabetes mellitus, n(%)	18 (24.0)
Hyperlipidemia, n (%)	21 (28.8)
Tobacco, n (%)	13 (17.1)
Atrial Fibrillation, n (%)	5 (6.8)
Angina Pectoris, n (%)	6 (8.1)
History of myocardial infarction, n (%)	4 (5.4)
History of heart failure, n (%)	3 (4.2)
Peripheral artery disease, n (%)	5 (6.8)
NIHSS at admission, median (IQR)	3 (1–4)
Systolic blood pressure, mmHg, mean (SD)	148.7 (22.0)
Diastolic blood pressure, mmHg, mean (SD)	77.4 (14.6)
Body mass index, kg/m^2^, mean (SD)	27.5 (5.1)

*SD* standard deviation, *IQR* interquartile range *NIHSS* National Institutes of Health Stroke Scale score

In a first step we focused on the measurement of LVEF being the main read-out parameter in our study. Of the 78 patients with two echocardiograms, reliable LVEF assessment was achieved in 73 patients, since ultrasound quality of both POC and standard echocardiography was insufficient in 5 patients; in these subjects, LVEF was assessed by eyeballing (i.e. <55% or ≥55%). In a second step, we investigated a series of other potentially treatment-relevant cardiac structures. Table 2 summarizes the quality of ultrasound conditions and results of echocardiographic measurements in POC and standard echocardiography.

**Table 2:** Point-of-care and standard echocardiographic data

	Point-of-care echocardiography	Standard echocardiography	Intraclass correlation coefficient (95% CI)	Agreement coefficient κ (95% CI)
Image quality, n (%)				
Parasternal				0.73 (0.61–0.86)
Good	35 (45.5)	40 (51.3)		
Moderate	22 (28.6)	20 (25.6)		
Poor	20 (26.0)	18 (23.1)		
Apical				0.65 (0.50–0.79)
Good	32 (42.1)	39 (50.0)		
Moderate	26 (34.2)	25 (32.1)		
Poor	18 (23.7)	14 (17.9)		
Subcostal				0.79 (0.67–0.91)
Good	38 (48.7)	41 (52.6)		
Moderate	28 (35.9)	24 (30.8)		
Poor	12 (15.4)	13 (16.7)		
Left ventricular ejection fraction, %, mean (SD)	60.8 (7.2)	62.3 (5.6)	0.82 (0.71–0.89)	
Left ventricular ejection fraction, n (%)				0.63 (0.40–0.85)
< 30%	0 (0.0)	0 (0.0)		
30–44%	3 (3.9)	1 (1.3)		
45–55%	10 (13.2)	7 (9.1)		
> 55%	63 (82.9)	69 (89.6)		
Not measurable, n	2	1		
Left ventricular end-diastolic wall thickness (septum), mm, mean (SD)	10.8 (1.7)	10.7 (1.7)	0.84 (0.76–0.90)	
Left ventricular end-diastolic wall thickness (posterior wall) mm, mean (SD)	10.5 (1.7)	10.0 (1.7)	0.85 (0.72–0.92)	
Left ventricular end-systolic diameter, mm, mean (SD)	33.7 (6.4)	32.9 (7.1)	0.93 (0.87–0.96)	
Left ventricular end-diastolic diameter, mm, mean (SD)	49.8 (5.5)	50.0 (5.5)	0.86 (0.79–0.91)	
Aortic valve maximal systolic flow velocity, m/s, mean (SD)	1.5 (0.4)	1.5 (0.4)	0.92 (0.88–0.95)	
Pericardial effusion, n (%)	0 (0.0)	0 (0.0)		0.00 (0.00–0.00)
Pleural effusion, n (%)	1 (1.3)	1 (1.3)		0.39 (−0.14–0.92)
Atrial septum aneurysm, n (%)	3 (3.9)	4 (5.1)		0.69 (0.39–0.98)
Heart rate during examination, 1/min, mean (SD)	69.8 (10.7)	69.7 (11.9)	0.67 (0.52–0.78)	

*CI* confidence interval, *SD* standard deviation

Interrater reliability was calculated between POC and standard echocardiography for all measured parameters. ICC ranged from 0.82 for LVEF to 0.93 for left ventricular end-systolic diameter (Table 2). Agreement coefficients for cardiac measurements ranged from 0.39 (pleural effusion) to 0.69 (atrial septum aneurysm), and for sonographic conditions from 0.65 (apical view) to 0.79 (subcostal view) (Table 2).

Given the study design and rationale as well as the LVEF-specific sample size estimation we restricted the calculation of further statistical values to LVEF as a marker for systolic function. Sensitivity of POC echocardiography for detecting systolic dysfunction was 100%, specificity 92.6%, and AUC 0.99 (95% CI, 0.96–1.00). Interrater reliability of the LVEF measurement by POC echocardiography between the two non-expert examiners was analyzed by an independent investigation of 18 patients. The ICC between the two raters for LVEF was 0.64 (95% CI 0.26–0.85).

## Discussion

This pilot study showed that POC echocardiographic assessment performed in the ambitious setting of a stroke unit by stroke unit staff using a portable device is feasible and achieves reliable results regarding selected key characteristics of cardiac dimensions and function.

Feasibility and reliability of POC echocardiography have been assessed in different settings before. A recent report underscores the feasibility and usefulness to establish POC echocardiography even during the medical studies [8]. Our results are in line with previous investigations reporting good correlations between POC and standard echocardiography for the quantification of cardiac structures and functions in patients with suspected cardiac abnormalities (e.g. congestive heart failure) treated in cardiology departments [8, 9]. Of note, in most studies ultrasound examiners were experienced cardiologists and/or sonographers [4, 9–11], whereas in our study non-cardiologists performed the POC examinations. Another group found only moderate correlations between POC and standard echocardiography, and discordant findings (mainly regarding wall motion abnormalities) in 27% of adult out-patients even when performed and interpreted by experienced operators [11].

To the best of our knowledge, we present the first report evaluating portable echocardiography in patients with acute ischemic stroke in the setting of a stroke unit. The majority of patients had neurological deficits and POC echocardiography had to be performed in regular hospital beds which made the examination particularly challenging. Nevertheless, we were able to demonstrate high precision for the detection of systolic dysfunction and structural cardiac abnormalities compared to standard echocardiography performed by expert sonographers under routine conditions.

POC echocardiography might be a useful tool for documenting relevant cardiac abnormalities, used to inform research studies regarding the underlying cause of stroke. The role of POC echocardiography regarding intraventricular thrombus or other life threatening findings could not be assessed as they were not prevalent in our patient collective. Nevertheless, in this pilot study, atrial septal aneurysms could be detected with high precision. Further studies to evaluate the reliability of detection of persistent foramen ovale via transthoracic POC echocardiography applying right heart contrast agent seem promising and might make standard echocardiography unnecessary in certain patients.

Some limitations of the current pilot study need to be addressed. As it was mainly designed for measurement of LVEF in the SICFAIL study, we did not include all potentially treatment-relevant cardiac parameters into the echocardiography protocol (e.g. detailed description of regional LV dysfunction or mitral valve analysis). The sample size estimation was calculated for measurement of LVEF as a marker of systolic function only. Therefore, despite high accuracy of POC measurements, low patient numbers with peculiar cardiologic findings (pericardial effusion *n* = 0, pleural effusion *n* = 1, atrial septum aneurysm *n* = 4, intracardiac thrombus *n* = 0) impede reliable conclusions regarding these conditions. Due to the monocentric design, generalization of our results to conditions outside of our hospital is limited. The echocardiography device was of high quality and of the same brand as the reference device; this might have improved comparability and reliability of measurements. The stroke unit setting (e.g. type of hospital beds, illumination of patient rooms) may also vary amongst institutions. In addition, potential selection bias cannot be excluded as severely handicapped patients could not provide informed consent.

## Conclusion

POC echocardiography is feasible in an acute stroke unit setting. Given the high accuracy for the detection of systolic dysfunction, it may be used for research purposes evaluating cardiac function after stroke. Its further clinical role as a screening tool for guiding individual patient treatment and management, such as fluid management in the acute situation, or for guiding secondary prevention, e.g. by detection of a cardiac source of embolism, which could render a standard echocardiogram unnecessary under certain conditions, needs to be established in larger prospective diagnostic studies with a sufficient number of patients with peculiar cardiologic findings.

## Additional file

Additional file 1: Figure S1. Illustration of the point-of-care ultrasound device (Vivid q, GE Healthcare, equipped with a M3S 1.5–4.0 MHz scanner). (DOCX 36 kb)

## Abbreviations

AUC: Area under curve
CI: Confidence interval
ICC: Intraclass correlation coefficient
IQR: Interquartile range
IS: Ischemic stroke
LVEF: Left ventricular ejection fraction
NIHSS: National Institutes of Health Stroke Scale score
POC: Point of care
SD: Standard deviation
SE: Standard echocardiography
SICFAIL: Stroke induced cardiac failure
SU: Stroke unit
WHO: World health organization
