# Supplementary material for: Feasibility and diagnostic accuracy of point-of-care handheld echocardiography in acute ischemic stroke patients – a pilot study
Source: BMC Neurol. 2017 Aug 11;17:159. doi: 10.1186/s12883-017-0937-8 (PMC5553778; doi:10.1186/s12883-017-0937-8)
Supplement: Additional file 1: Figure S1. — Illustration of the point-of-care ultrasound device (Vivid q, GE Healthcare, equipped with a M3S 1.5–4.0 MHz scanner). (DOCX 36 kb) [file 12883_2017_937_MOESM1_ESM.docx]

**Feasibility and Diagnostic Accuracy of Point-of-Care Handheld Echocardiography in Acute Ischemic Stroke Patients – a Pilot Study**

**Supplemental Figure 1**


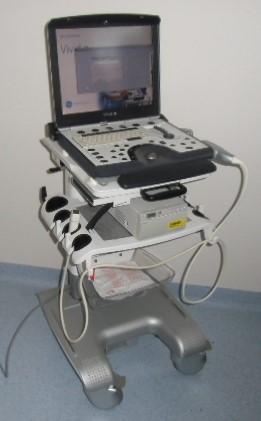


**Supplemental Figure 1:** Illustration of the point-of-care ultrasound device (Vivid q, GE Healthcare, equipped with a M3S 1.5-4.0 MHz scanner)
